# Taxonomic and Trophic Groups Mediate Latitudinal Variation in Saproxylic Beetle Species Richness and Body Size Across Western Palaearctic Oak Forests

**DOI:** 10.1002/ece3.71574

**Published:** 2025-06-16

**Authors:** M. Franzen, N. Jansson, M. Avci, A. Brin, H. Brustel, J. Budka, J. Buse, G. Carpaneto, S. Chiari, L. Cizek, M. Coskun, J. Dagley, P. M. Hammond, E. Micó, T. Öncul Abacigil, T. Pavlicek, J. Schlaghamersky, P. Sebek, A. Sverdrup‐Thygeson, S. Vural Varli, L. Westerberg, I. Wilde, A. Zauli, P. Milberg

**Affiliations:** ^1^ IFM Biology, Division of Ecology Linköping University Linköping Sweden; ^2^ Department of Biology and Environmental Sciences Linnaeus University Kalmar Sweden; ^3^ Department of Forest Entomology and Protection, Faculty of Forestry Süleyman Demirel University Isparta Türkiye; ^4^ École D'ingénieurs de Purpan Toulouse France; ^5^ Department of Botany and Zoology, Faculty of Science Masaryk University Brno Czech Republic; ^6^ Department for Ecological Monitoring Research and Species Protection Seebach Germany; ^7^ Department of Science University of Rome Rome Italy; ^8^ Institute of Entomology Biology Centre of the Czech Academy of Sciences České Budějovice Czech Republic; ^9^ Department of Biology, Faculty of Science and Literature Adıyaman University Adıyaman Türkiye; ^10^ Essex Wildlife Trust, Abbotts Hall‐Joan Elliot Building Essex UK; ^11^ Natural History Museum London UK; ^12^ Research Institute CIBIO (Centro Iberoamericano de la Biodiversidad) University of Alicante Alicante Spain; ^13^ Olive and Olive Processing Technology Department, Vocational High School of Edremit Balıkesir University Balıkesir Türkiye; ^14^ Institute of Evolution University of Haifa Haifa Israel; ^15^ Department of Ecology and Natural Resource Management Norwegian University of Life Sciences Ås Norway; ^16^ Faculty of Science and Arts Balikesir University Balikesir Türkiye; ^17^ Nottinghamshire Wildelife Trust, The Old Ragged School Nottingham UK

**Keywords:** biodiversity, body size, latitudinal gradient, macroecology, oak forests, saproxylic beetles

## Abstract

We examined latitudinal gradients in species richness and body size of saproxylic beetles across 28 veteran oak forest sites spanning from Israel to Norway. Focusing on 425 species from 11 taxonomic families and five trophic groups, we tested three hypotheses to elucidate: (i) family‐specific richness responses to latitude, (ii) trophic mediation of richness patterns, (iii) whether body size follows Bergmann‐like clines. We found significant family‐level variations in richness–latitude relationships. These non‐uniform patterns highlight the importance of taxonomic resolution in capturing macroecological diversity gradients. Body size analyses revealed significant latitude associations, indicating that both phylogenetic constraints and trophic group modulate latitudinal size patterns among saproxylic beetles. Taken together, our findings emphasize that macroecological patterns in saproxylic beetles are shaped by a synthesis of phylogenetic history and functional traits. Conservation strategies should, therefore, account for family‐level and trophic‐group heterogeneity, particularly as climate warming and shifting resource distributions may differentially affect lineages with distinct thermoregulatory and life‐history constraints. These results underscore the need for taxon‐specific approaches when predicting and managing biodiversity in changing oak forest ecosystems.

## Graphical abstract

In this study, we examined latitudinal gradients in species richness and body size among 425 saproxylic beetle species from 28 oak forest sites spanning from Israel to Norway. Our analyses show that taxonomic identity and trophic group both mediate latitudinal patterns, with several families displaying mid‐latitude diversity peaks and saproxyloxylophagous species exhibiting a pronounced mid‐latitude size maximum. These findings highlight the importance of phylogenetic and functional trait frameworks for understanding macroecological variation in forest ecosystems.

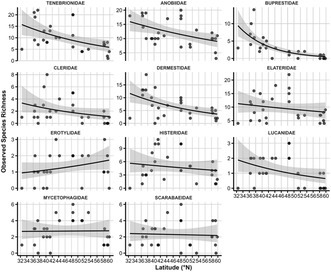

## 1 Introduction

Latitudinal gradients in species richness and body size represent one of ecology's most enduring and widely studied patterns (Gaston and Blackburn 2000; Hillebrand 2004; Willig et al. 2003). Since Wallace's and Darwin's era, the observation that biodiversity tends to peak in tropical regions has catalyzed the development of over 30 hypotheses attempting to explain this phenomenon (Hawkins et al. 2003; Mittelbach et al. 2007). These gradients manifest across diverse taxonomic groups and spatial scales, serving as the foundation for broad macroecological theories about the spatial distribution of biodiversity (Chown and Gaston 2010; Jablonski and Hunt 2006). However, increasing evidence suggests that deviations from classic latitudinal diversity patterns are common, particularly among ectothermic invertebrates (Kindlmann et al. 2007; Vázquez and Stevens 2004). Unlike vertebrates, which generally display more consistent diversity declines toward the poles, insects often exhibit complex, nonlinear responses to latitude (Lima et al. 2008; Sheldon 2019). These idiosyncratic patterns reflect the interacting influences of evolutionary history, climatic variables, biotic interactions, and resource availability (Beck et al. 2012; Sundqvist et al. 2013). Recent investigations have revealed that taxonomic resolution, functional traits, and ecological specialization can profoundly modulate latitudinal responses, leading to clade‐specific patterns that may deviate substantially from general trends (Diniz Filho et al. 2023; Guedes et al. 2025).

Alongside species richness, body size represents a fundamental trait exhibiting predictable geographical variation across diverse taxa (Gaston and Chown 2013; Olalla‐Tárraga et al. 2010). Bergmann's rule—originally proposing that endothermic animals tend to be larger in colder environments—has stimulated extensive research on body size‐climate relationships (Blackburn et al. 1999; Meiri and Dayan 2003). While this pattern often holds for mammals and birds through thermoregulatory mechanisms, its applicability to ectotherms remains contentious, with numerous studies reporting inconsistent or even contrary patterns (Angilletta and Dunham 2003; Watt et al. 2010). The mechanistic foundations of size clines in ectotherms likely differ fundamentally from those in endotherms, potentially involving developmental plasticity, seasonal time constraints, and resource availability rather than heat conservation per se (Angilletta et al. 2004; Blanckenhorn and Demont 2004). Insects, in particular, display remarkable heterogeneity in their body size responses to latitudinal gradients (Chown and Gaston 2010; Shelomi 2012). The temperature‐size rule—suggesting that ectotherms develop faster but mature at smaller sizes in warmer environments—offers one potential explanation for insect size clines (Atkinson 1994; Kingsolver and Huey 2008). However, this relationship is frequently complicated by countervailing selective pressures related to voltinism, seasonal synchrony, starvation resistance, and predation risk (Chown and Klok 2003; Horne et al. 2015; Kingsolver and Huey 2008).

Taxonomic identity frequently modifies latitudinal responses in arthropods, reflecting different evolutionary lineages and ecological niches (Buckley et al. 2010; Condamine et al. 2012). Studies across diverse insect groups reveal that higher taxonomic levels (e.g., orders, families) often show distinctive latitudinal patterns in both diversity and trait distributions (Boyero et al. 2011; Schowalter 2022; Wong et al. 2019). These differences likely stem from clade‐specific ecological adaptations, dispersal capacities, and historical biogeography (Buckley et al. 2010; Jablonski 2017). Among beetles specifically, family‐level analyses have demonstrated considerable heterogeneity in latitudinal richness and body size patterns, highlighting the importance of taxonomic resolution in macroecological research (Heino et al. 2015, 2019). Such taxonomic mediation of latitudinal patterns underscores the value of multi‐family studies that can disentangle general trends from lineage‐specific responses (Hernández Fernández et al. 2022; Rollinson and Rowe 2018).

Beyond taxonomy, functional traits like trophic position can profoundly influence species' responses to environmental gradients (Keppeler et al. 2020; McGill et al. 2006). Trophic groups (e.g., xylophagous, mycetophagous, and saprophagous) can shape distribution patterns as resource availability and climatic factors differ across latitudes (Hagge et al. 2019; Micó 2018). For instance, predatory species may track prey distributions, while detritivores might respond more directly to decomposition rates and resource turnover, which themselves vary with climate (Martins et al. 2023; Swift et al. 1979). Among saproxylic beetles, trophic specialisation can affect species' thermal tolerances, habitat specificity, and dispersal capabilities—all factors potentially influencing geographical distributions (Seibold et al. 2015; Wende et al. 2017). A growing body of work suggests that examining functional traits in tandem with taxonomic identity can clarify the mechanisms underlying latitudinal diversity gradients (Boyero et al. 2015; Lamanna et al. 2014). This integrated approach may be particularly valuable for saproxylic beetles, where resource specialisation and feeding strategies potentially mediate species' responses to broad‐scale environmental gradients (Sebek et al. 2012; Seibold and Thorn 2018).

### 1.1 Saproxylic Beetles as Model Organisms

Saproxylic beetles play crucial roles in nutrient cycling, wood decomposition, and forest ecology, making them ecologically important indicators (Speight 1989). Their remarkable diversity, with thousands of species dependent on deadwood habitats, encompasses multiple feeding guilds, body sizes, and life histories (Ulyshen and Šobotník 2018). This functional diversity, combined with their documented sensitivity to environmental conditions, renders saproxylic assemblages valuable model systems for testing macroecological hypotheses (Bouget et al. 2013; Grove 2002; Lachat et al. 2006). Many saproxylic beetle species exhibit specific microhabitat requirements, limited dispersal capabilities, and varying thermal tolerances, potentially generating complex and taxonomically structured responses to latitudinal gradients (Buse et al. 2007; Franzén et al. 2025; Janssen et al. 2016).

Their sensitivity to climate and resource availability, combined with the diverse functional roles among different families, makes saproxylic beetles ideal for testing latitudinal macroecological hypotheses (Müller et al. 2015; Ulyshen 2016). Changes in temperature and precipitation across latitudes can directly affect beetle development, survival, and resource quality (Gossner et al. 2013; Seibold and Thorn 2018). Additionally, the distribution and characteristics of deadwood habitats—including decay rates, fungal colonisation, and microclimate—change predictably with latitude, potentially driving parallel shifts in beetle assemblages (Heilmann‐Clausen et al. 2014; Ulyshen et al. 2018). Oak‐associated saproxylic assemblages, in particular, present a tractable system due to the broad geographic range and long evolutionary history of oak‐dominated forests (Ranius et al. 2024; Vodka et al. 2009). Oak forests extend across the Western Palearctic region, from Mediterranean to boreal zones, providing a consistent host substrate that minimises the confounding effects of tree species identity on beetle distributions (Milberg et al. 2014; Sirami et al. 2008). The characteristic veteran oaks found across this range support particularly species‐rich and specialised beetle assemblages, offering an excellent opportunity to investigate broad‐scale biodiversity patterns (Ranius and Jansson 2000; Sverdrup‐Thygeson et al. 2010).

### 1.2 Hypotheses

In this study, we test three hypotheses regarding latitudinal patterns in saproxylic beetle diversity:

1. Saproxylic beetle richness exhibits distinct, family‐specific latitudinal patterns, potentially peaking at mid‐latitudes due to optimal resource availability and climate (Hawkins et al. 2003; Rahbek 1995). If so, we expect that both linear and nonlinear terms for latitudewill be significant.
2. Trophic groups and beetle families exhibit latitudinal patterns due to differing resource requirements and climate tolerances (Seibold et al. 2015; Ulyshen and Šobotník 2018).
3. Body size varies with latitude, but differs among families (Chown and Gaston 2010; Shelomi 2012).

## 2 Methods

### 2.1 Study Area

**FIGURE 1:**
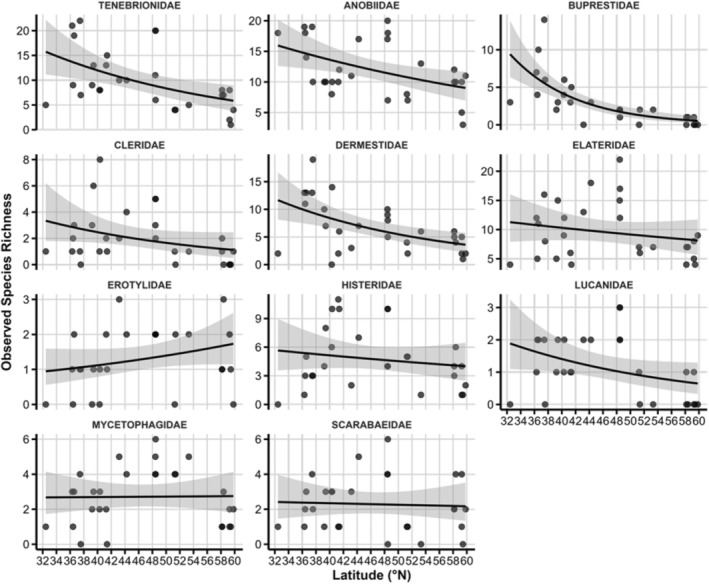
Latitudinal gradients in observed beetle species richness across families based on the negative binomial GLMM results. The shaded area represents the 95% CI.

We studied 28 sites with veteran oaks in 10 countries in Europe, Turkey, and Israel (Figure 1a). Ten mature *Quercus* spp. individuals were selected at each site based on characteristics associated with high saproxylic beetle diversity. All selected trees contained cavities. Different *Quercus* species occur at these sites, ranging in elevation from 10 to 1500 m a.s.l. Previous studies suggest limited host‐tree specificity among saproxylic beetles in deciduous trees (Milberg et al. 2014), suggesting that *Quercus* species differences are unlikely to influence beetle assemblages significantly. The average circumference of the studied trees ranged from 175 to 612 cm. The minimum distance between any two trees selected at a site was 20 m, with the maximum distance being 8300 m.

### 2.2 Study Sites and Sampling

Beetles were sampled within a single season per site, starting between 10 March and 19 May and concluding between 10 August and 4 October, from 1994 to 2012. The duration of the sampling period varied from 3 to 6 months, tailored to align with the active flight period of the beetles, which decreases in length with increasing latitude. A flight interception trap was installed on each of the 10 selected trees, ideally positioned in front of or near a cavity entrance, at heights ranging from 1 to 7 m to capture flying beetles. The traps were designed with 30 cm × 50 cm (width × height) transparent plastic windows attached to a container filled with ethylene glycol, water, and detergent. The traps were checked monthly. The authors and experts for the respective groups/families identified beetles at the species level. Following (Speight 1989) definition of saproxylic species, the final dataset focused exclusively on saproxylic and oak‐associated beetle species from 11 selected families: Anobiidae (106 species), Buprestidae (28 species), Cleridae (16 species), Dermestidae (55 species), Elateridae (67 species), Erotylidae (10 species), Histeridae (32 species), Lucanidae (6 species), Mycetophagidae (11 species), Scarabaeidae (16 species), and Tenebrionidae (78 species). These families were selected based on the reliable identification capabilities within the study's scope and available taxonomic expertise. Fourteen species collected during this study have since been described as new to science (Mazur et al. 2013; Novák et al. 2011; Platia et al. 2011), while 13 species are awaiting taxonomic revisions and formal descriptions. Unidentified species were classified as morphospecies based on morphological characters and are undergoing ongoing taxonomic revision (Göktepe et al. 2023). Taxonomic classification adhered to the standards outlined in Fauna Europaea (de Jong 2016).

### 2.3 Body Size Measurements

Body size was measured as body length (from the top of the head to the end of the abdomen, to the nearest 0.1 mm) using callipers. An average of three measures from male beetles per species was calculated.

### 2.4 Larval Diet Classification (Trophic Groups)

Each species was classified into one of five larval trophic groups following Bouget et al. (2005): mycetophagous (37 species), saprophagous (99 species), sapro‐xylophagous (96 species), xylophagous (78 species) and zoophagous (115 species). The classification was updated using current knowledge from the authors and experts for species whose biology remains poorly documented or whose classification has changed since Stokland and Meyke (2008). Mycetophagous beetles feed on fungi that colonize dead or decaying wood. Their activity facilitates the breakdown of lignin and cellulose, contributing significantly to nutrient cycling within forest ecosystems (Boddy 2001). These beetles often have specific associations with fungal species, shaping fungal distributions and influencing forest decomposition dynamics (Biedermann and Vega 2020). Saprophagous beetles consume decaying organic matter, which is critical in soil formation and health, by processing various plant detritus and animal remains (Swift et al. 1979). Sapro‐xylophagous beetles bridge the ecological functions of mycetophagous and saprophagous guilds by feeding on decaying wood and associated fungi or microorganisms. Their adaptable feeding habits make them essential for wood decomposition and nutrient cycling (Ulyshen et al. 2018; Ulyshen and Šobotník 2018). Xylophagous beetles are primary decomposers of dead wood, initiating its breakdown and facilitating nutrient cycling and soil development in forest ecosystems (Speight 2005). Zoophagous beetles, as predators, regulate invertebrate populations. Their adaptations, such as enhanced sensory organs and strong mandibles, highlight their specialized role in forest food webs (Crowson 2013; Jabin et al. 2004). Each trophic group contributes uniquely to decomposition processes and nutrient cycling, supporting the food webs and ecological dynamics of old‐growth oak forests (Bauhus et al. 2018; Bouget et al. 2005).

### 2.5 Statistical Analyses

All analyses were conducted in R version 4.3.2 (R Core Team 2024). Plots were generated with *ggplot2* version 3.5.1 (Wickham and Wickham 2007).

### 2.6 Species Richness Estimation

To address incomplete sampling, we employed an interpolation/extrapolation approach with the iNext package in R version 3.0.1 (Hsieh et al. 2024). This produced coverage‐based Hill species richness estimates. Species richness was thus summarized by family to compare patterns of sampling completeness per taxonomic family per site. To avoid bias, we included all site‐family combinations, even those with zero detections.

### 2.7 Richness Models

We investigated latitudinal patterns in observed and estimated beetle species richness via negative binomial generalized linear mixed models (GLMMs) in *glmmTMB* version 1.1.10 (Brooks et al. 2023). Observed richness was the total species count, and estimated richness was the rounded Hill numbers for each site–taxonomic family combination. We specified a negative binomial distribution to account for overdispersion. Model predictors included linear and quadratic terms for latitude. The site was included as a random effect to account for repeated measures at each site. Separate models were run for each beetle family.

### 2.8 Body Size Models

We tested body size–latitude relationships using two separate GLMMs in *glmmTMB*. In the first model, the response variable was the mean log_10_‐transformed body size of each family at each site, with linear and quadratic latitude terms. In the second model, we examined mean log_10_‐transformed body size by the trophic group at each site, again with linear and quadratic latitude terms. One model was run separately for each beetle family.

## 3 Results

We surveyed 28 sites spanning Israel to Norway, documenting 425 saproxylic beetle species across 11 families and five trophic groups. Peak local richness ranged from 12 species (at the northernmost site) to 99 species (at the central latitude), suggesting strong geographical variation potentially linked to latitude and associated climatic gradients.

**FIGURE 2:**
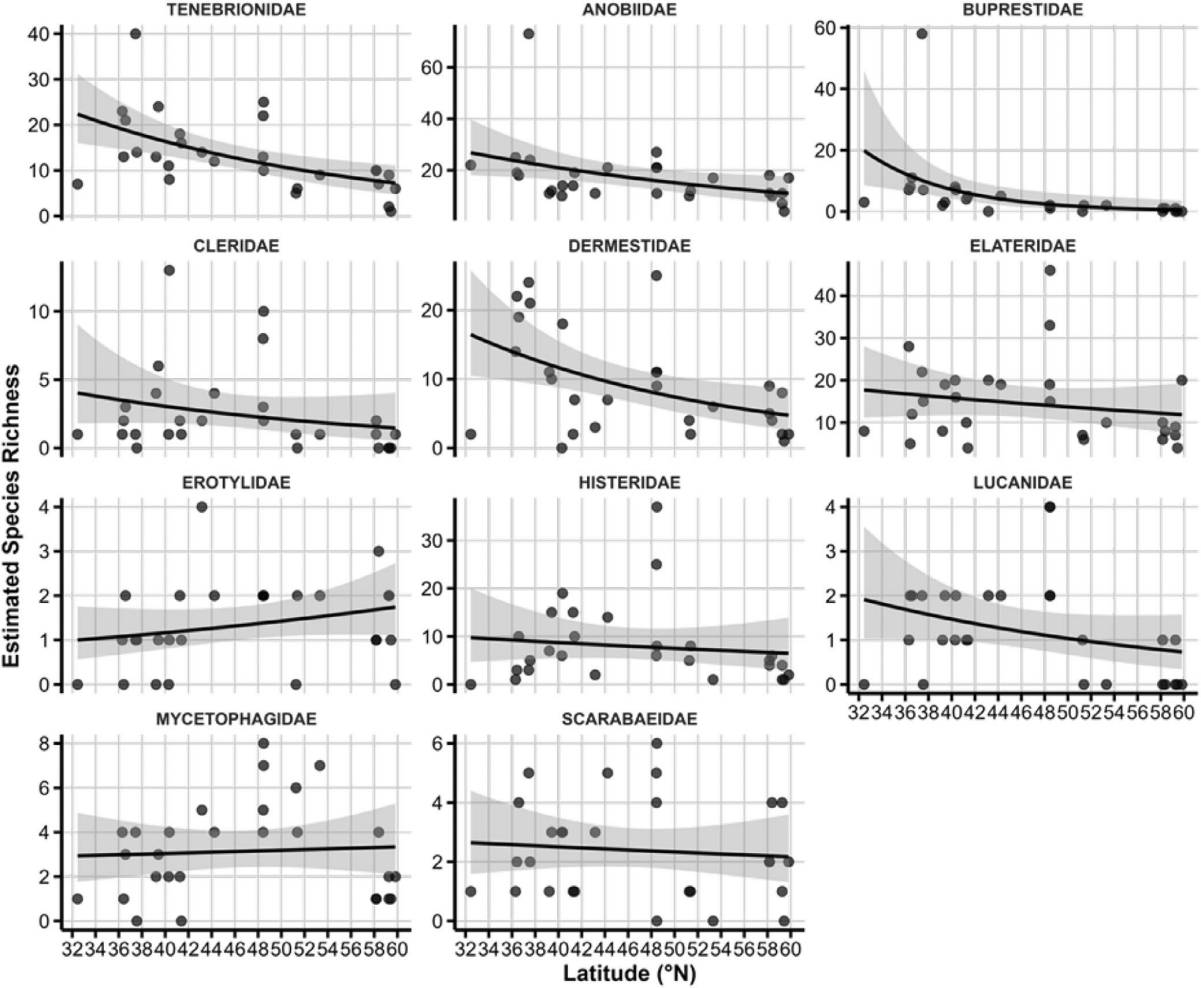
Latitudinal gradients in estimated beetle species richness across families derived from the negative binomial GLMM results. The shaded area represents the 95% CI.

Species richness in relation to linear latitude coefficients was significantly negative in eight of the eleven families (*p* < 0.05), with the steepest declines in Buprestidae (−23.3 ± 4.1) and Cleridae (−12.4 ± 3.9). Significant quadratic terms in Elateridae, Lucanidae, Mycetophagidae, and Histeridae indicate unimodal or U‐shaped responses, i.e., richness peaks or troughs at intermediate latitudes. Estimated richness patterns closely mirrored the observed data without being statistically significant, except for the linear term for Dermestidae (Table 1, Figures 1 and 2).

**TABLE 1:** Latitudinal effects on beetle species richness across taxonomic families based on negative binomial GLMMs. Results show (a) observed and (b) estimated richness. Significant *p*‐values are in bold.

Beetle family	Latitude effects	(a) Observed richness	SE	*p*	(b) Estimated (Hill) richness	SE	*p*
		Est.			Est.		
Anobiidae	Linear	−3.94	1.57	**0.0118**	−0.77	0.85	0.363
Anobiidae	Quadratic	−0.37	1.54	0.8092	0.62	0.73	0.397
Buprestidae	Linear	−23.3	4.09	**< 0.001**	−4.42	3.02	0.143
Buprestidae	Quadratic	−1.62	3.14	0.6070	−1.6	1.13	0.157
Cleridae	Linear	−12.39	3.93	**0.0016**	−1.26	1.99	0.526
Cleridae	Quadratic	−10.12	3.39	**0.0028**	2.17	1.36	0.112
Dermestidae	Linear	−5.92	2.99	**0.0479**	−2.29	0.56	**< 0.001**
Dermestidae	Quadratic	−4.29	3.03	0.1564	−0.58	0.53	0.278
Elateridae	Linear	−2.19	1.48	0.1389	−0.83	0.98	0.400
Elateridae	Quadratic	−6.79	1.47	**< 0.001**	−1.44	0.99	0.145
Erotylidae	Linear	0.99	3.38	0.7685	0.12	1.18	0.918
Erotylidae	Quadratic	−6.93	3.45	**0.0448**	0.12	1.21	0.919
Histeridae	Linear	−0.34	2.57	0.8953	0.88	0.81	0.278
Histeridae	Quadratic	−8.4	2.65	**0.0015**	0.45	0.88	0.607
Lucanidae	Linear	−10.08	4.21	**0.0166**	0.22	3.63	0.952
Lucanidae	Quadratic	−15.05	3.62	**< 0.001**	0.11	2.3	0.962
Mycetophagidae	Linear	2.27	2.59	0.3806	0.49	0.81	0.542
Mycetophagidae	Quadratic	−8.39	2.6	**0.0013**	−1.75	1.02	0.086
Scarabaeidae	Linear	−4.62	3.38	0.1706	1.09	0.9	0.225
Scarabaeidae	Quadratic	−1.68	3.34	0.6151	−1.22	0.8	0.125
Tenebrionidae	Linear	−6.88	1.8	**< 0.001**	0.01	1.13	0.992
Tenebrionidae	Quadratic	−2.77	1.78	0.1207	−0.2	1.21	0.869

### 3.1 Body Size Variation Across Latitude

**FIGURE 3:**
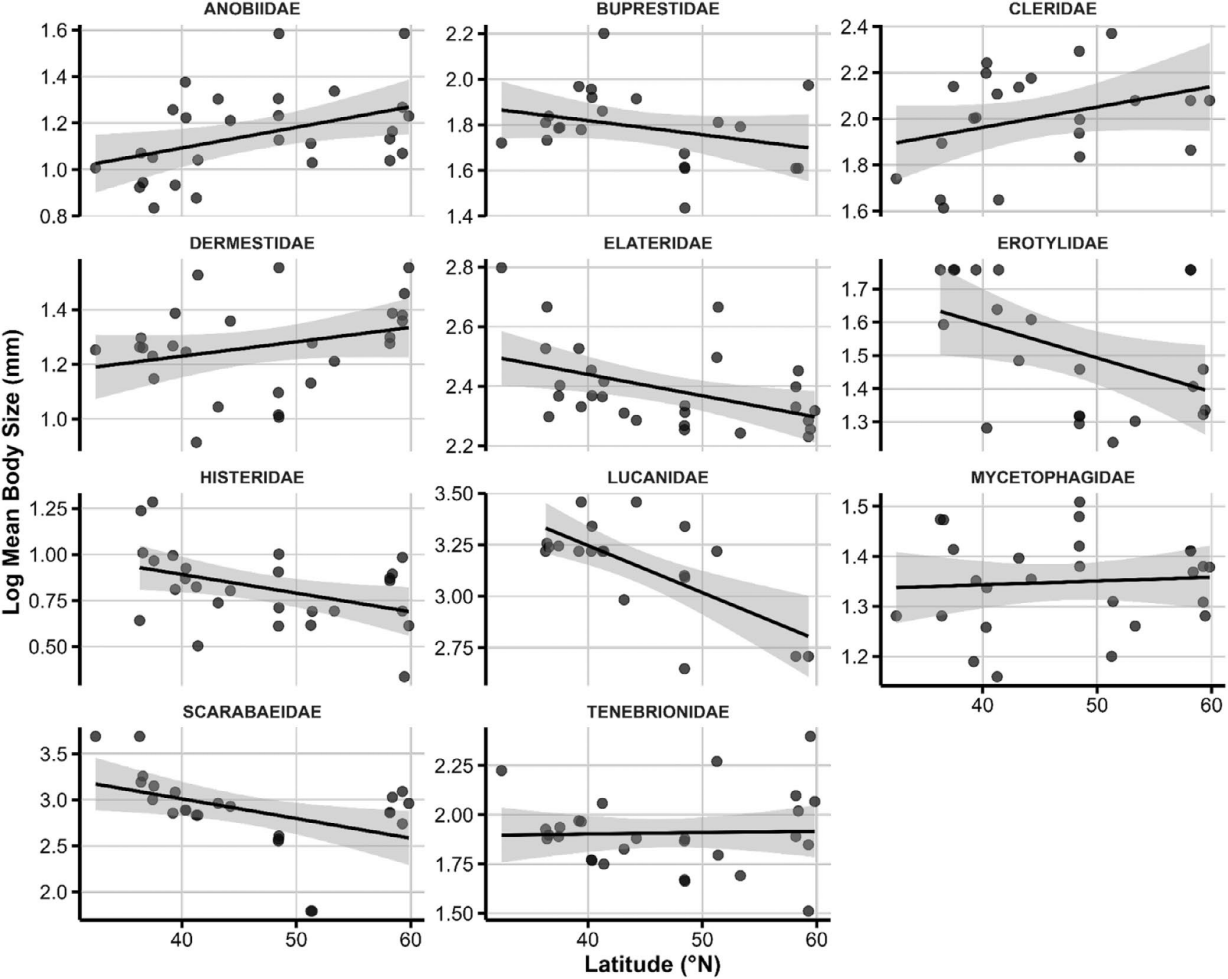
Latitudinal gradients in average body size of different saproxylic beetle families. The shaded area represents the 95% CI.

Five families show significant latitudinal declines in log_10_ body length: Elateridae (−4.59 ± 1.32), Erotylidae (−1.87 ± 0.70), Histeridae (−1.87 ± 0.51), Lucanidae (−11.30 ± 4.98) and Scarabaeidae (−21.03 ± 3.61; all *p* < 0.05). Anobiidae exhibits a modest but significant increase (1.56 ± 0.59), whereas the remaining families display non‐significant slopes (Table 2, Figure 3). Six families possess significant second‐order terms, revealing non‐linear size responses. Cleridae and Lucanidae have negative quadratic coefficients, implying mid‐latitude maxima. Conversely, Elateridae, Erotylidae, Histeridae, and Scarabaeidae have positive quadratic terms, consistent with mid‐latitude minima (Table 2, Figure 3).

**TABLE 2:** Body size variation across beetle families with latitude based on linear mixed‐effects models.

Beetle family	Latitude effects	Estimate	SE	*p*
Anobiidae	Linear	1.56	0.59	**0.0083**
Anobiidae	Quadratic	−0.88	0.59	0.1367
Buprestidae	Linear	−1.62	1.15	0.1587
Buprestidae	Quadratic	−0.65	1.15	0.5739
Cleridae	Linear	2.04	1.3	0.1182
Cleridae	Quadratic	−3.62	1.3	0.0054
Dermestidae	Linear	0.84	0.56	0.1326
Dermestidae	Quadratic	0.67	0.56	0.2326
Elateridae	Linear	−4.59	1.32	**< 0.001**
Elateridae	Quadratic	2.82	1.32	**0.0330**
Erotylidae	Linear	−1.87	0.7	**0.0073**
Erotylidae	Quadratic	1.97	0.7	**0.0047**
Histeridae	Linear	−1.87	0.51	**< 0.001**
Histeridae	Quadratic	1.04	0.51	**0.0432**
Lucanidae	Linear	−11.3	4.98	**0.0232**
Lucanidae	Quadratic	−14.59	4.98	**0.0034**
Mycetophagidae	Linear	0.03	0.37	0.9365
Mycetophagidae	Quadratic	−0.17	0.37	0.6534
Scarabaeidae	Linear	−21.03	3.61	**< 0.001**
Scarabaeidae	Quadratic	27.94	3.61	**< 0.001**
Tenebrionidae	Linear	−0.03	1.22	0.9798
Tenebrionidae	Quadratic	2.01	1.22	0.1000

*Note:* Body size values are log_10_‐transformed. Significant *p*‐values (*p* < 0.05) are in bold.

Abbreviation: SE, standard error.

**FIGURE 4:**
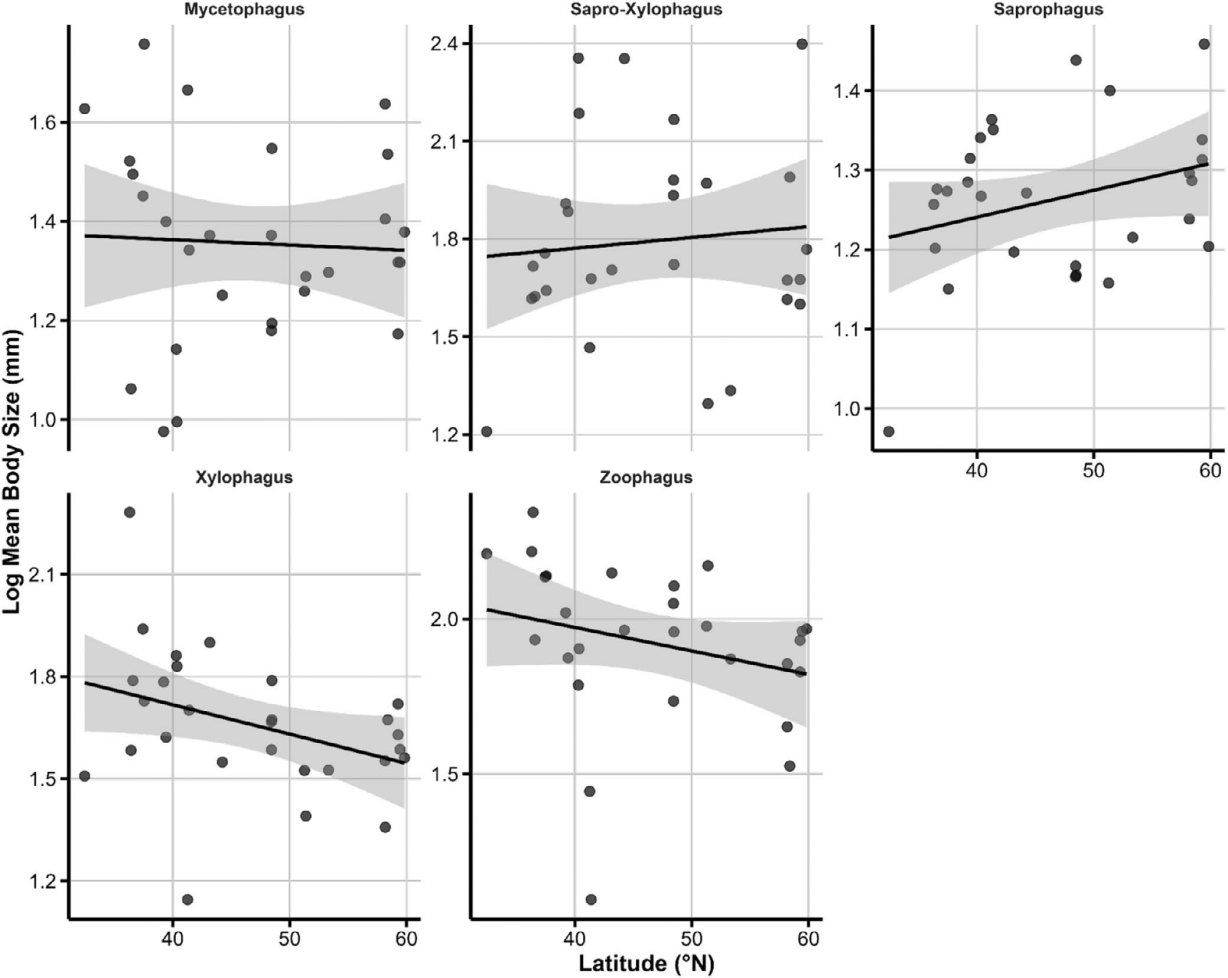
Latitudinal gradients in average body size across trophic groups of saproxylic beetles. The shaded area represents the 95% CI.

Xylophagous species displayed the steepest decline in log_10_ body length with increasing latitude (linear = −6.55 ± 2.04, *p* = 0.0013). Zoophagous species showed a more moderate but still significant decrease (linear = −3.22 ± 1.49, *p* = 0.0305). All other groups exhibited non‐significant linear terms (Table 3, Figure 4). Quadratic curvature approached significance in sapro‐xylophagous (quadratic = −3.91 ± 2.18, *p* = 0.0728) and saprophagous beetles (quadratic = −0.80 ± 0.44, *p* = 0.0680), suggesting possible mid‐latitude maxima, but confidence intervals overlapped zero. No quadratic effect was detected in the two groups that had significant linear trends (Table 3, Figure 4).

**TABLE 3:** Body size variation across beetle trophic groups with latitude based on linear mixed‐effects models.

Beetle family	Latitude effects	Estimate	SE	*p*
Mycetophagus	Linear	−0.73	0.79	0.3593
Mycetophagus	Quadratic	1.23	0.79	0.1200
Sapro‐xylophagus	Linear	1.15	2.18	0.5963
Sapro‐xylophagus	Quadratic	−3.91	2.18	0.0728
Saprophagus	Linear	0.19	0.44	0.6618
Saprophagus	Quadratic	−0.8	0.44	0.0680
Xylophagus	Linear	−6.55	2.04	**0.0013**
Xylophagus	Quadratic	1.18	2.04	0.5644
Zoophagus	Linear	−3.22	1.49	**0.0305**
Zoophagus	Quadratic	1.36	1.49	0.3602

*Note:* Body size values are log_10_‐transformed. Significant *p*‐values (*p* < 0.05) are in bold.

Abbreviation: SE, standard error.

## 4 Discussion

### 4.1 Family‐Specific Latitudinal Patterns in Species Richness

Our first hypothesis—that saproxylic beetle richness exhibits distinct family‐specific latitudinal patterns—is strongly supported by the significant associations between species richness and latitude. Several families, notably Buprestidae, Cleridae, Elateridae, Erotylidae, Mycetophagidae, and Scarabaeidae, showed mid‐latitude peaks in species richness, whereas others displayed more linear responses or no significant trends. This heterogeneity underscores that saproxylic beetle assemblages do not conform to a universal latitudinal diversity gradient (Kouki et al. 2012; Ulyshen and Šobotník 2018).

Mid‐latitude richness peaks may stem from optimal combinations of temperature, precipitation, and resource availability in temperate oak forests, paralleling mid‐domain patterns in other taxa (Beck et al. 2012; Jetz and Rahbek 2002; Rahbek 1995). For specialised families such as Elateridae and Mycetophagidae, the milder climatic conditions and ample deadwood resources found at temperate latitudes could facilitate higher reproductive success while avoiding the shortened growing seasons of northern sites (Müller et al. 2015; Seibold and Thorn 2018). Family‐specific responses also illustrate that higher taxonomic resolution is vital for uncovering macroecological patterns (Boyero et al. 2011; Vodka et al. 2009). Analyses grouping all saproxylic beetles under a single umbrella would obscure clade‐specific signals arising from divergent evolutionary histories and ecological specialisations (Moura and Jetz 2021).

### 4.2 Body Size Variation Across Latitude: Taxonomic and Trophic Influences

Our third hypothesis—that body size distributions follow predictable latitudinal clines varying by family—is supported by the significant associations between body size and latitude. Families exhibited pronounced linear or curvilinear size–latitude relationships, consistent with findings that insects vary widely in adherence to Bergmann's rule (Chown and Gaston 2010; Shelomi 2012). Phylogenetic lineage and clade‐specific physiological constraints appear to modulate how individual families respond to latitudinal temperature gradients (Chown and Klok 2003; Kaspari et al. 2024).

Body size differed markedly among trophic guilds, but latitude modified those patterns only in a subset of groups. Xylophagous beetles showed the steepest latitudinal decline, and zoophagous beetles a moderate but still significant decrease, whereas fungivorous, saprophagous, and sapro‐xylophagous guilds displayed non‐significant linear trends. Quadratic curvature approached significance in the two sapro‐based guilds, hinting at possible mid‐latitude optima, but confidence intervals overlapped zero. These results suggest that wood‐feeding and predatory strategies make body size more sensitive to thermal or developmental constraints than detrital or fungivorous strategies (Potapov et al. 2019; Ulyshen 2016). Thus, ecological niche modulates body‐size responses to latitude, yet the mixed guild‐specific outcomes reinforce the conclusion that no single mechanism—whether temperature‐size rules, seasonal time limitation, or resource availability—fully explains the observed gradient; phylogenetic constraints remain an important overlay on trophic effects (Atkinson 1994; Blanckenhorn and Demont 2004; Brown et al. 2004).

### 4.3 Conservation Implications

Our findings highlight family‐specific and trophic group‐specific responses to latitude, reinforcing the idea that veteran oak forests are not ecologically uniform across the western Palearctic. Mid‐latitude regions hosting diversity peaks in certain families may function as “hotspots” deserving enhanced conservation efforts (Müller et al. 2014; Vodka et al. 2009). Such spatially explicit management is crucial because generalized, one‐size‐fits‐all conservation strategies risk overlooking the distinct ecological requirements of families exhibiting different richness and body size responses (Diniz Filho et al. 2023). Moreover, climate change could alter latitudinal thermal regimes and resource distributions, pushing some beetle lineages toward suboptimal conditions or forcing shifts in body size and phenology (Gardner et al. 2011; Seibold and Thorn 2018). Predictive models incorporating evolutionary background and trophic strategies may better forecast how saproxylic beetle assemblages will reorganize under future climates.

## 5 Limitations and Future Directions

Although our dataset spans diverse oak habitats from Israel to Norway, our focus on a single host‐tree genus may limit broader generalizations to other forest ecosystems. Longitudinal extensions, including additional host species and finer climatic data, could clarify how abiotic and biotic factors intersect to shape latitudinal patterns (Chown and Klok 2003). Incorporating phylogenetic analyses would help disentangle the roles of shared ancestry versus convergent adaptations. Lastly, mechanistic experiments on temperature‐ or resource‐driven body size constraints could further elucidate how multiple ecological drivers interact to produce the clade‐specific patterns we observed (Mähn et al. 2023; Weeks et al. 2022).

## 6 Conclusion

Our results demonstrate that latitudinal gradients in saproxylic beetle richness and body size are taxon‐specific, driven by both phylogenetic (family‐level) and trophic (feeding‐guild) factors. This multi‐faceted variation emphasizes the need to integrate taxonomic resolution, functional traits, and evolutionary history when examining macroecological patterns across broad spatial scales. Informed by this nuanced perspective, targeted conservation actions and predictive models can be more accurately tailored to conserve beetle diversity in the face of ongoing environmental change (Guedes et al. 2025).

## Author Contributions

**M. Franzen:** conceptualization (equal), data curation (equal), formal analysis (equal), funding acquisition (equal), visualization (equal), writing – original draft (equal), writing – review and editing (equal). **N. Jansson:** investigation (equal), validation (equal). **M. Avci:** investigation (equal), methodology (equal). **A. Brin:** data curation (equal), formal analysis (equal), project administration (equal). **H. Brustel:** data curation (equal), software (equal). **J. Budka:** visualization (equal). **J. Buse:** data curation (equal), resources (equal). **G. Carpaneto:** validation (equal), visualization (equal). **S. Chiari:** formal analysis (equal). **L. Cizek:** software (equal). **M. Coskun:** visualization (equal). **J. Dagley:** validation (equal). **P. M. Hammond:** investigation (equal). **E. Micó:** project administration (equal). **T. Öncul Abacigil:** writing – original draft (equal). **T. Pavlicek:** conceptualization (equal). **J. Schlaghamersky:** resources (equal). **P. Sebek:** project administration (equal). **A. Sverdrup‐Thygeson:** writing – original draft (equal). **S. Vural Varli:** writing – review and editing (equal). **L. Westerberg:** resources (equal), software (equal). **I. Wilde:** formal analysis (equal). **A. Zauli:** investigation (equal). **P. Milberg:** writing – original draft (equal), writing – review and editing (equal).

## Ethics Statement

This study was conducted following ethical guidelines and principles.

## Conflicts of Interest

The authors declare no conflicts of interest.

## Supporting information


Data S1.

## Data Availability

All data are provided as Supporting Information.
